# Current Status and Future Strategies to Increase Secondary Metabolite Production from Cyanobacteria

**DOI:** 10.3390/microorganisms8121849

**Published:** 2020-11-24

**Authors:** Yujin Jeong, Sang-Hyeok Cho, Hookeun Lee, Hyung-Kyoon Choi, Dong-Myung Kim, Choul-Gyun Lee, Suhyung Cho, Byung-Kwan Cho

**Affiliations:** 1 Department of Biological Sciences and KAIST Institutes for the BioCentury, Korea Advanced Institute of Science and Technology, Daejeon 34141, Korea; mist@kaist.ac.kr (Y.J.); graysky@kaist.ac.kr (S.-H.C.); 2 Institute of Pharmaceutical Research, College of Pharmacy, Gachon University, Incheon 21999, Korea; hklee@gachon.ac.kr; 3 College of Pharmacy, Chung-Ang University, Seoul 06911, Korea; hykychoi@cau.ac.kr; 4 Department of Chemical Engineering and Applied Chemistry, Chungnam National University, Daejeon 34134, Korea; dmkim@cnu.ac.kr; 5 Department of Biological Engineering, Inha University, Incheon 22212, Korea; leecg@inha.ac.kr

**Keywords:** cyanobacteria, photosynthesis, secondary metabolites, metabolic engineering, synthetic biology, systems biology, genome-scale model

## Abstract

Cyanobacteria, given their ability to produce various secondary metabolites utilizing solar energy and carbon dioxide, are a potential platform for sustainable production of biochemicals. Until now, conventional metabolic engineering approaches have been applied to various cyanobacterial species for enhanced production of industrially valued compounds, including secondary metabolites and non-natural biochemicals. However, the shortage of understanding of cyanobacterial metabolic and regulatory networks for atmospheric carbon fixation to biochemical production and the lack of available engineering tools limit the potential of cyanobacteria for industrial applications. Recently, to overcome the limitations, synthetic biology tools and systems biology approaches such as genome-scale modeling based on diverse omics data have been applied to cyanobacteria. This review covers the synthetic and systems biology approaches for advanced metabolic engineering of cyanobacteria.

## 1. Introduction

Cyanobacteria are oxygenic photosynthetic bacteria that can produce various secondary metabolites. Given the ability to utilize sunlight and atmospheric carbon dioxide (CO_2_) as a part of the renewable photosynthetic process, cyanobacteria are considered sustainable bioproduction hosts [1]. A number of secondary metabolites naturally synthesized by cyanobacteria, such as carotenoids, phycocyanins, and squalene, are used in the pharmaceutical, cosmetic, and healthcare industries [2,3,4]. In addition, owing to their rapid growth and increased scope for engineering, multiple efforts have been made to utilize cyanobacteria as production hosts for valuable biochemicals by introducing heterologous pathways [5,6].

While continuous development has been reported in metabolic engineering strategies for producing biochemicals in bacterial hosts, the synthetic biology approach accelerated the development by providing diverse genetic parts and engineering tools. For other model platforms such as *Escherichia coli*, there is an abundant catalog of genetic parts including synthetic promoters and ribosome binding sites (RBSs), which have been successfully introduced to improve gene expression in heterologous pathways [7]. However, owing to the lack of genetic parts for pathway engineering in cyanobacteria, application of metabolic engineering tools is limited [8]. Thus, development of various tools for pathway engineering and subsequent engineering strategies are required for industrial-scale production of target compounds in cyanobacteria.

With the recent progress in systems biology, genome-wide information of diverse layers such as the genome, transcriptome, translatome, proteome, metabolome, and interactome are being constantly accumulated [9]. Massive amounts of data formed the basis for establishment and development of an in silico genome-scale model (GEM) [10]. It is expected that the application of system-level approaches with the integration of omics data and GEM would address the existing limitations of cyanobacterial engineering. The current review not only describes the value-added secondary metabolites produced by cyanobacteria and current metabolic engineering approaches for their production but also introduces the synthetic and systems biology approach for further development.

## 2. Secondary Metabolite Production by Cyanobacteria

Bacteria produce two kinds of metabolites: primary metabolites essential for survival and secondary metabolites required for auxiliary purposes, such as stress responses, defense mechanisms, metal carrying, and signaling [11]. Secondary metabolites include terpenes, alkaloids, polyketides (PKs), non-ribosomal peptides (NRPs), and ribosomally synthesized and post-translationally modified peptides (RiPPs), which are produced via biosynthetic gene clusters (BGCs). BGCs are clusters of genes positioned in approximate proximity to each other for the production and processing of a compound. Cyanobacteria, being rich in BGCs, are capable of producing diverse secondary metabolites for various purposes, including toxins for defenses or protectants for relieving photodamage and oxidative stress (Table 1).

### 2.1. Prediction of Biosynthetic Gene Clusters (BGCs) in Cyanobacterial Genomes

**Figure 1:**
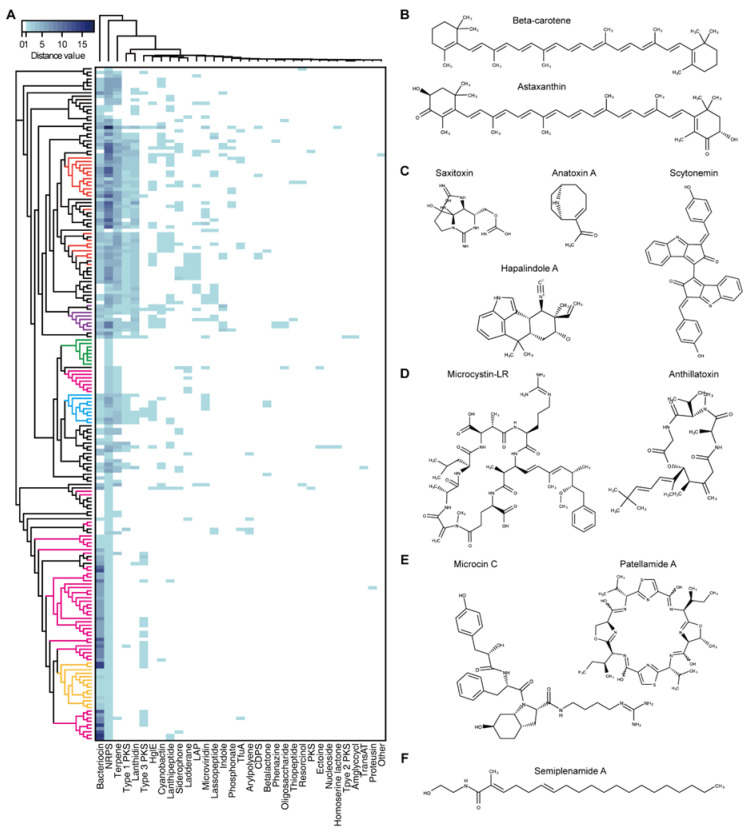
Cyanobacterial secondary metabolites. (**A**) Heatmap of the predicted cyanobacterial secondary metabolite biosynthstic gene clusters (BGCs). The left-most phylogenetic tree is constructed by up-to-date bacterial core gene (UBCG) phylogenetic analysis of the 196 cyanobacterial complete genome sequences. The evolutionary distances were provided by UBCG and plotted by RAxML [80,81]. The tree is not to scale. Red: *Nostoc*, purple: *Calothrix*, green: *Synechocystis*, pink: *Synechoccus*, blue: *Microcystis*, and yellow: *Prochlorococcus*. (**B**–**F**) Molecular structures of cyanobacterial secondary metabolites. (**B**) Terpenes, (**C**) alkaloids, (**D**) polyketides (PKs), non-ribosomal peptides (NRPs), (**E**) RiPPs, and (**F**) fatty acid amide. Abbreviations; NRPS, non-ribosomal peptide synthetase; HglE, heterocyst glycolipid synthase; LAP, linear azol(in)e-containing peptide; TfuA, ribosomally synthesized peptide antibiotic trifolitoxin; CDPS, cyclodipeptide synthase-based tRNA dependent peptide; PKS, polyketide synthase; Amglyccycl, aminoglycosides/aminocyclitols; TransAT, trans-acyltransferase type I PKS.

To investigate the secondary metabolites produced by cyanobacteria, 196 complete genome sequences of cyanobacteria available at the National Center for Biotechnology Information (NCBI) genome portal were inspected for BGCs using antiSMASH [74]. Thirty-three different types of BGCs were identified. The 196 complete genome sequences of cyanobacteria used in the BGC search were arranged according to the phylogenetic tree. The heatmap representing the numbers of each type of BGC found in each cyanobacterium showed that the cyanobacteria from the same genera had similar classes and numbers of BGCs (Figure 1A). It was evident that a single genome contained several BGCs with multiple occurrences. In particular, there were cyanobacteria with large number of bacteriocin, terpene, and non-ribosomal peptide synthetase (NRPS) BGCs, which accounted for 74.4% of the total predicted BGCs (*n* = 2119). For example, it was predicted that the genome of *Moorea producens* PAL-8-15-08-1 carries 18 NRPS BGCs. The most widely distributed BGC was the terpene BGC, which was found in all cyanobacteria except for two species (*Limnospira fusiformis* SAG 85.79 and *Nodularia spumigena* UHCC 0039). Terpene is essential for photosynthetic organisms. Undetected terpene BGCs in the two species could have resulted from the deviations in the BGC search criteria of antiSMASH. The 33 BGCs were classified according to their structural and functional similarities to the following categories: terpene, indole, PK synthase (PKS)/NRPS (type 1, 2, 3 PKSs, NRPS, cyclodipeptide synthase-based tRNA-dependent peptide, resorcinol, and siderophore), RiPP (bacteriocin, lanthidin, linear azole-containing peptide, microviridin, lasso peptide, cyanobactin, thiopeptide, trifolitoxin, proteusin, and lanthipeptide), lipid/saccharide/nucleoside (heterocyst glycolipid synthase, ladderane, arylpolyene, aminoglycoside/aminocyclitol, oligosaccharide, and nucleoside), and others (phosphonate, phenazine, ectoine, β-lactone, and homoserine lactone).

### 2.2. Terpenes

Terpene is a family of compounds with varying structures that occupies a large proportion of the natural products [75]. Terpenes are mainly produced by plants or fungi, as well as the bacterial species via mevalonate (MVA) pathway or methylerythritol-phosphate (MEP) pathway using acetyl-CoA or glyceraldehyde 3-phosphate and pyruvate as substrates [76]. While MVA and MEP pathways are mutually exclusive in most organisms, cyanobacteria mainly utilize the MEP pathway, using substrates generated during photosynthesis. The MEP pathway produces isomeric 5-carbon compounds, isopentyl pyrophosphate (IPP), and dimethylallyl pyrophosphate (DMAPP), which are further condensed into geranyl pyrophosphate (GPP), the building block in terpene biosynthesis. From the GPP, terpenes of varying structures can be generated. Terpenes conduct various cellular processes necessary for survival, such as the ubiquinone in the electron transport chain associated with cellular respiration, chlorophyll, carotenoids, and plastoquinones in photosynthetic processes, and hopanoids in cell membrane biosynthesis and stability (Figure 1B) [77]. In particular, photosynthetic cyanobacteria contain a wide variety of carotenoids. Most of the genome-sequenced cyanobacteria have β-carotene BGC. Production of other carotenoids, such as zeaxanthin and nostoxanthin are dependent on the presence of carotenogenesis pathway connected to β-carotene [3,78]. The terpene compounds, including the carotenoids obtained from cyanobacteria are of industrial value owing to their various applications. For example, β-carotene, astaxanthin, and canthaxanthin are used as color additives or animal feeds. Phycocyanin exhibits anti-oxidant, anti-inflammatory, neuroprotective, and hepatoprotective effects [2,13,79].

### 2.3. Alkaloids

Alkaloids comprise various nitrogen containing compounds that are produced from diverse organisms, including fungi, plants, bacteria, and animals. Alkaloids produced by cyanobacteria often show toxic characteristics. For example, the anatoxin-a produced by species of the *Anabaena* genera is a neurotoxin that binds irreversibly to nicotinic acetylcholine receptors causing paralysis or even death in fish and mammals (Figure 1C) [31]. Anatoxin-a is also categorized as a PK, which is synthesized by PKS [82]. Another well-known example, saxitoxin, blocks the sodium (Na^+^) channels in shellfish and induces paralytic shellfish poisoning in humans on consumption of saxitoxin-accumulated seafood. The chemical derivatives carrying the indole rings are classified as indole alkaloids. They are biosynthesized using tryptophan as a precursor. Cyanobacterial indole alkaloids have diverse functions. For example, the hapalindole synthesized from cyanobacteria *Hapalosiphon fontinalis* exhibits antibacterial, anti-tuberculosis, and anticancer activities [83]. In addition, the scytonemin produced by *Scytonema* sp. renders photoprotective effects to the cyanobacterial cells by absorbing the harmful ultraviolet (UV)-A radiation [84].

### 2.4. Polyketides/Non-Ribosomal Peptide/Lipopeptides/Siderophores

PKS and NRPS are representatives of enzymes responsible for the biosynthesis of secondary metabolites in various organisms. Enzymes of these classes consists of at least three essential modular domains that facilitate chain elongation and modification [85]. First, the catalytic domain binds to and activates the building block, which then is transferred to the carrier protein domain. Second, the carrier protein domain loads the activated building block to the growing PK/NRP chain it holds. Third, the other catalytic domain catalyzes the bond formation between the growing chain and the newly loaded building block. PKS and NRPS differ in their use of precursors for the building block. While PKS utilizes malonyl-CoA or methylmalonyl-CoA, the NRPS uses proteinogenic and non-proteinogenic amino acid monomers. In addition, there are cases wherein compounds are synthesized via the PKS–NRPS hybrid system. A well-known example could be microcystin, the BGC of which contains two PKS, single PKS–NRPS, and three NRPS [42,86]. Microcystin produced from various cyanobacterial species belonging to the genus Microcystis, Nostoc, Planktothrix, and Anabaena, shows hepatotoxic activity in humans (Figure 1D). Various other toxins synthesized by the PKS, NRPS, or PKS–NRPS hybrid system includes lyngbyatoxin, apratoxin, and aplysiatoxin.

The NRPS includes lipopeptides owing to their lipid linked peptide structures synthesized by a combination of lipid tails and amino acids. Examples of lipopeptides include antillatoxin and carmabin from *M. producens*, and lyngbyabellin from *M. bouillonii* (Figure 1D). Antillatoxin and lyngbyabellin show neurotoxic activity and cytotoxicity, and carmabin exhibit anti-malarial activity. Siderophores are included in the NRPS-produced compounds. Iron is essential for bacterial survival. However, since it exists in an insoluble form in the environment, some bacteria have evolved to facilitate iron uptake by producing small molecules with high affinity to ferric iron, called siderophores.

### 2.5. Ribosomally Synthesized and Post-Translationally Modified Peptides

RiPP is a class of secondary metabolites that includes, as its name depicts, ribosomally synthesized and post-translationally modified peptides. Post-translational modifications include leader peptide hydrolysis, cyclization, and disulfide bond formation. RiPP BGC generally consists of a short precursor peptide with an *N*-terminal leader and a *C*-terminal core sequence, and post-translational modification (PTM) enzymes [87,88]. The PTM enzymes shape the linear peptide by several modifications that provide structural and functional diversity to the mature scaffold. Compounds that were previously classified as lanthipeptide, lasso peptide, microviridin, cyanobactin, and microcin are now re-classified under RiPP, which have a broad range of bioactivities such as protease inhibition, cytotoxicity, signaling, anti-cancer, and anti-human immunodeficiency virus (anti-HIV) (Figure 1E) [87]. For example, microviridin, which was first isolated from *M. viridis*, is a serine protease inhibitor, and patellamide A produced by *Prochloron didemni* has moderate cytotoxicity [62].

### 2.6. Lipids/Saccharides/Nucleosides/Others

Lipids, saccharides, and nucleosides are generally categorized as primary metabolites. However, there are exceptions, when they are considered as secondary metabolites instead of primary metabolites. For example, besarhanamide A and semiplenamide exhibiting toxicity against brine shrimp are fatty acid amides isolated from *M. producens* and *Lyngbya semiplena*, respectively (Figure 1F) [66,89]. It is known that cyanobacterium *Cyanothece* sp. 113 can produce up to 22 g/L of polysaccharide, which exceeds the producing ability of eukaryotic microalgae, such as *Dunaliella salina* [90,91]. Polysaccharides are generally used as stabilization or thickening agents for emulsions. In some cases, they are used as bioactive compounds owing to their antitumor, antiviral, antibacterial, anti-inflammatory, and immunostimulatory properties [92,93,94,95]. Toyocamycin and tubercidin are both anti-fungal nucleoside chemicals isolated from *Tolypothrix tenuis* [96]. In addition, a small number of phosphonate, phenazine, ectoine, and β-lactone BGC were also detected.

## 3. Engineering Cyanobacteria for Industrial Production of Secondary Metabolites

**Figure 2:**
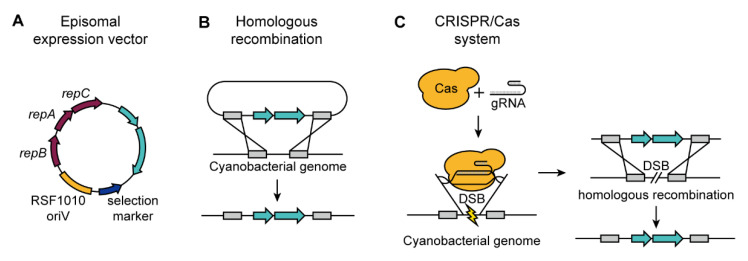
Genetic engineering tools. (**A**) Homologous recombination method using the recombination system in cyanobacteria. (**B**) RSF1010-derived vectors are self-replicating vectors used in episomal expression vector system. (**C**) CRISPR/Cas system utilizes Cas endonuclease to generate double-strand break to the gRNA-escorted loci inducing homologous recombination. Abbreviation; CRISPR, clustered regularly interspaced short palindromic repeat; gRNA, guide RNA; DSB, double-strand break.

Engineering efforts have been made to increase the production of industrially important cyanobacterial natural compounds. The model cyanobacteria such as *Synechocystis* sp. PCC 6803 and *Synechococcus elongatus* PCC 7942 are often used as engineering hosts for the increased ease of genetic manipulation. The biosynthetic pathways of other cyanobacteria are adopted to these model species by heterologous expression for production of value-added compounds. In addition to the cyanobacterial natural products, cyanobacteria have also been identified as a suitable heterologous platform for the production of biofuels, such as ethanol, butanol, and 2,3-butanediol [5,97,98,99]. Episomal expression using a self-replicating vector is a popular method for introducing foreign genetic elements in other organisms such as *E. coli*. Compared to chromosomal integration through homologous recombination, the episomal expression is more advantageous owing to its higher expression level [100]. In addition, the genome polyploidy of cyanobacteria can cause problems in the natural recombination process by reversing the engineered genome copies back to the original sequence, resulting in poor engineering efficiency and a laborious selection process. However, in cyanobacteria, there are minimal options for vector systems; the only self-replicating vector origin currently available for application is RSF1010 (Figure 2A). Thus, chromosomal integration or deletion through homologous recombination is the most dominant method used in cyanobacteria to increase the production of natural compound or heterologous metabolites (Figure 2B). Recently developed clustered regularly interspaced short palindromic repeat (CRISPR)/Cas is an effective genome engineering tool that can target specific loci to generate a double-strand break, and thus it can solve the low engineering efficiency problem in polyploids (Figure 2C) [101]. Additionally, the repurposed CRISPR/Cas system, namely the CRISPR interference (CRISPRi), can repress the gene expression without nucleic acid strand excision, avoiding lethality caused by knock-out of essential genes.

### 3.1. Heterologous Expression for Cyanobacterial Secondary Metabolite Production

Genetic manipulation tools explained above have been applied in cyanobacteria to increase the production of secondary metabolites (Table 2). In recent years, multiple studies have targeted terpenes, such as squalene and limonene. Squalene has widespread applications in the healthcare, cosmetics, and pharmacological fields, and it is produced from several eukaryotes as well as the cyanobacteria, such as *Phormidium autumnale* [19,102]. However, squalene production from cyanobacteria is not sufficient for the industrial-scale production demands. Metabolic engineering efforts were made in cyanobacterium model, *S. elongatus* PCC 7942 [103,104,105]. *S. elongatus* PCC 7942 has the methylerythritol phosphate (MEP) pathway to biosynthesize diphosphate (IPP) and dimethylallyl diphosphate (DMAPP) from CO_2_. DMAPP is converted to farnesyl diphosphate (FPP), a substrate for squalene biosynthesis, by FPP synthase (*ispA*). Heterogenous genes, including 1-deoxy-d-xylulose-5-phosphate synthase (*dxs*), isopentenyl diphosphate isomerase (*idi*), and *ispA* were introduced into the *S. elongatus* PCC 7942 genome by homologous recombination to increase the intracellular concentration of FPP. Next, a squalene synthase (*SQS*) was also introduced by homologous recombination, resulting in a maximum of 5.0 mg/L/OD_730nm_ squalene production [103]. The titer was further increased to 12.0 mg/L/OD_730nm_ by constructing a fusion protein of *SQS* with *cpcB1*, which encodes the β-subunit of phycocyanin and is highly expressed under the strong endogenous *cpcB1* promoter [104]. Recently, CRISPRi was applied to the squalene-producing *S. elongatus* PCC 7942 strain to repress two essential genes, *acnB* and *cpcB2* encoding aconitase and phycocyanin β-subunit, respectively, resulting in an improved squalene production [105]. The results of these previous studies suggest that there is sufficient potential to improve the production of target compounds in cyanobacteria through the discovery of new potent promoters or the selection of additional engineering targets.

Genetic manipulation tools explained above have been applied in cyanobacteria to increase the production of secondary metabolites (Table 2). In recent years, multiple studies have targeted terpenes, such as squalene and limonene. Squalene has widespread applications in the healthcare, cosmetics, and pharmacological fields, and it is produced from several eukaryotes as well as the cyanobacteria, such as *Phormidium autumnale* [19,102]. However, squalene production from cyanobacteria is not sufficient for the industrial-scale production demands. Metabolic engineering efforts were made in cyanobacterium model, *S. elongatus* PCC 7942 [103,104,105]. *S. elongatus* PCC 7942 has the methylerythritol phosphate (MEP) pathway to biosynthesize diphosphate (IPP) and dimethylallyl diphosphate (DMAPP) from CO_2_. DMAPP is converted to farnesyl diphosphate (FPP), a substrate for squalene biosynthesis, by FPP synthase (*ispA*). Heterogenous genes, including 1-deoxy-d-xylulose-5-phosphate synthase (*dxs*), isopentenyl diphosphate isomerase (*idi*), and *ispA* were introduced into the *S. elongatus* PCC 7942 genome by homologous recombination to increase the intracellular concentration of FPP. Next, a squalene synthase (*SQS*) was also introduced by homologous recombination, resulting in a maximum of 5.0 mg/L/OD_730nm_ squalene production [103]. The titer was further increased to 12.0 mg/L/OD_730nm_ by constructing a fusion protein of *SQS* with *cpcB1*, which encodes the β-subunit of phycocyanin and is highly expressed under the strong endogenous *cpcB1* promoter [104]. Recently, CRISPRi was applied to the squalene-producing *S. elongatus* PCC 7942 strain to repress two essential genes, *acnB* and *cpcB2* encoding aconitase and phycocyanin β-subunit, respectively, resulting in an improved squalene production [105]. The results of these previous studies suggest that there is sufficient potential to improve the production of target compounds in cyanobacteria through the discovery of new potent promoters or the selection of additional engineering targets.

Besides terpenes, a xanthophyll carotenoid called astaxanthin has been gaining significant attention in the healthcare field owing to the anti-inflammatory and antioxidant properties elucidated in human cells [106]. Astaxanthin production was enhanced through the engineering of *Synechocystis* sp. PCC 6803 [107]. First, the core biosynthetic genes, β-carotenoid ketolase (*crtW*) and β-carotene hydroxylase (*crtZ*) were integrated for astaxanthin production. The promoter combinations with diverse strength were tested for expressions of *crtW* and *crtZ*, because their relative expression level is known to be critical to produce astaxanthin in *E. coli* [107]. However, astaxanthin production was detected only when the super-strong promoter Pcpc560 was used for both genes, indicating that other tested endogenous promoters (PnirA, PpetE, and PrnpB) were not sufficient to express those genes. To test the relative expression level of the two genes, a promoter pool with more varied strength, including the stronger promoter than Pcpc560, was required. By using Pcpc560 for *crtZ* expression and a pea promoter PsbA for *crtW* expression, which showed two-fold higher activity than Pcpc560, resulting in improved production of astaxanthin. Further, based on liquid chromatography-mass spectrometry (LC-MS) metabolomics data, fructose-1,6-/sedoheptulose 1,7-bisphosphate (FBP/SBPase), which is involved in the Calvin–Benson–Bassham cycle, was found as an additional engineering target and overexpressed with episomal expression vector. Then, heterologous *dxs* and *ispA* gene was introduced into *Synechocystis* sp. PCC 6803 genome, and the engineered strain was finally able to produce astaxanthin of 29.6 mg/g dry cell weight, the highest level in the currently known engineered strain.

### 3.2. Heterologous Expression for Biofuel Production

Along with the naturally produced cyanobacterial secondary metabolites, cyanobacteria also serve as an attractive platform for diverse heterologous biochemical production. For instance, cyanobacterial genome engineering was performed to use *S. elongatus* PCC 7942 as a host for producing 2,3-butanediol, a biochemical building block for plasticizers, liquid fuel additives, and industrial solvents [5]. In this study, homologous recombination was used for the integration of galactose-proton symporter (*galP*), glucose-6-phosphate dehydrogenase (*zwf*), and 6-phosphogluconate dehydrogenase (*gnd*) into neutral sites, and phosphoribulokinase (*prk*) and RuBisCO subunits (*rbcLXS*) into the *cp12* site, resulting in a maximum production of 12.6 g/L of 2,3-butanediol. In addition, in *Synechocystis* sp. PCC 6803, metabolic engineering was performed to enhance the biofuel production [99]. To improve ethanol production in the pyruvate decarboxylase (*pdc*)-inserted ethanol-producing *Synechocystis* sp. PCC 6803 strain, pyruvate dehydrogenase complex subunit (*odhB*) was repressed by CRISPRi. Repression of citrate synthase (*gltA*) in the phosphoketolase (*xfpk*) and acetoacetyl-CoA synthase (*nphT7*)-inserted n-butanol-producing strain increased *N*-butanol production.

Isoprene is a plant-derived building block, mainly used in the manufacturing of synthetic rubber. While most of the current synthetic rubber production depends on the petrochemical source, there have been efforts made to increase the cyanobacterial production of isoprene directly from CO_2_. On introducing the isoprene synthase (*ispS*) obtained from various plants into the *Synechocystis* sp. PCC 6803 genome by homologous recombination, a maximum of 4.3 mg/L/h isoprene production rate was achieved with the aid of *dxs* and *idi* overexpression [6]. The heterologous inducible promoter Ptrc or endogenous promoters Pcpc and PpsbA2 were tested for expression of diverse *ispS*, and as a result, expression of *Eucalyptus globulus ispS* with Ptrc promoter showed the highest isoprene production. As demonstrated in various studies, cyanobacteria can produce industrially valuable biomaterials by utilizing light and CO_2_. Therefore, cyanobacteria have the potential to be developed into an eco-friendly and economical photoautotrophic biofactory.

### 3.3. Improvement of Photosynthetic Efficiency

The value-added biochemicals that we have discussed are all products of photosynthesis. Thus, enhancement of the photosynthetic efficiency is crucial for supplying sufficient energy and reducing power for productivity increment. Some of the strategies for improving photosynthesis include expansion of the absorption spectra to capture more light energy, downsizing of the antenna to increase high illumination tolerance, and optimization of the electron transport chain. Furthermore, efficient use of photosystem-generated energy is another strategy that can be achieved by enhancing carbon fixation or reducing carbon loss [137].

### 3.4. Current Limitations in Engineering Cyanobacteria

Until now, we have presented studies showing the potential of cyanobacteria in producing various metabolites and that continuous engineering efforts can enhance the native and non-native metabolite production from cyanobacteria. However, despite the proposed and demonstrated potential as a production host, the production levels in cyanobacteria are not compatible with those in model organisms such as *E. coli* or *Saccharomyces cerevisiae*. In the case of *E. coli*, which is most widely used engineering host with a lot of information about genetic features and metabolic network, it is easy to apply knowledge-based engineering approaches such as enzyme structure modification, feedback inhibition removal, and precursor pool or cofactor level increasement [138]. In addition, a high-throughput screening technique through random mutagenesis is applicable using well-developed screening systems in *E. coli*. On the other hand, since cyanobacteria is a photoautotroph, more complex energy generation and distribution, and redox state should be considered when manipulating the metabolic network, and thus a more systematic insight is required. Additionally, when engineering multiple targets in the metabolic pathway, it is difficult to fine-tune the relative expression levels of the genes due to the lack of available bioparts such as neutral site, promoter, and RBS. In order to overcome these limitations, it is essential to systematically understand the complex metabolic network within the cell and to develop various genetic tools by genome-scale screening of native promoters and RBSs or constructing synthetic bioparts. In recent studies, a systematic approach through genome-scale modeling (GEM) has been successfully applied to engineering cyanobacteria [124,125]. For more effective and efficient engineering of cyanobacteria, the systematic approach should be further advanced.

## 4. Advanced Engineering Approaches through Synthetic and Systems Biology

**Figure 3:**
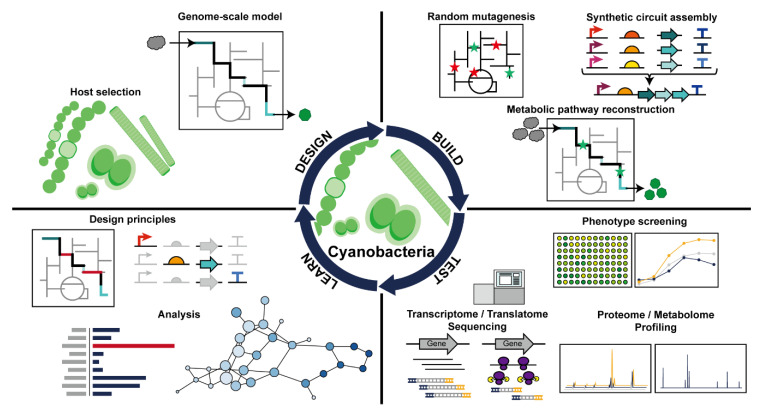
Schematic representation of design–build–test–learn cycle in cyanobacteria.

Synthetic biology involves development of genetic parts, combination design to fulfill the desired function, and application of the combined tool into an organism. Quantification and standardization of the genetic parts represented by promoters, RBS, untranslated region (UTR) sequences, and terminator sequences are critical for proper employment of synthetic biology. Systems biology deals with the living system as an interactive network more than just a collection of reductive components. Therefore, understanding of the organism as a system is required for precise designing of the synthetic biology tools, and the introduction of synthetic biology tools into an organism affects the system, making the two biological approaches inseparable. The general synthetic and systems biology research flow is represented as the design–build–test–learn cycle (Figure 3). In the design step, the host for metabolite production is selected, and the biosynthesis pathway is designed using prior knowledge. Then, in the build step, a bioproduction host is engineered using either random, rational, or both methods. The constructed strain may undergo various tests for data generation. The data are then analyzed to produce and update the understanding of the bioproduction system. Systems and synthetic biology as an integrative approach, assisted the engineering of various organisms, including cyanobacteria.

### 4.1. Synthetic Biology

Development of the genetic parts is critical for applying synthetic biology to metabolic engineering of cyanobacteria. While in other model species (e.g., *E. coli*), genetic parts such as promoters and RBS with varying strengths are available, there has been a significant lack of information and diversity concerning the cyanobacterial genetic parts. Currently used genetic parts are cyanobacterial endogenous promoters (P_psbA_ and P_cpc_) and *E. coli* origin promoters (P_trc_, P_BAD_, P_lac_, and P_nrsB_) [139]. Promoters currently used in cyanobacteria are cataloged in previous literature [140]. However, to expand the promoter selection pool, several P_T7_ derivatives, P_psbA_* derivatives, P_trc_-based hybrid, and synthetic promoters have been developed and tested in *Synechocystis* sp. PCC 6803 [141,142]. Recently, a mutant promoter library from two popular promoters, P_psbA_ and P_cpc_ of *S. elongatus* PCC 7942, were generated to achieve promoters of varying strength [143]. A collection of 48 unique promoters were validated in three additional *S. elongatus* strains, expanding the cyanobacterial synthetic biology toolbox. The RBSs for cyanobacterial gene expression are mostly wild-type RBS associated with native promoter, or RBS of highly expressed genes such as *psbA2* and *rbcL* [144]. In addition, synthetic RBSs from BioBrick Registry of standard biological parts, and a newly designed RBS based on *Synechocystis* sp. PCC 6803 genome sequence (RBS*) are also used in several studies [145]. Recently, research has been conducted to diversify the RBS types used in cyanobacterial engineering. Twenty types of native RBS from *Synechocystis* sp. PCC 6803 were additionally identified, and 13 RBSs were rationally designed based on the known strong RBS sequences [142,146].

Riboswitches are another class of bioparts operated based on their RNA structures. A riboswitch comprises of an aptamer and an expression part. While the aptamer directly binds to a corresponding small molecule, the expression part regulates gene expression post-transcriptionally by causing structural changes in accordance with the small molecule binding. In *S. elongatus* PCC 7942, the operation of the synthetic theophylline riboswitch confirmed that it could control translation initiation [147]. Recently, this riboswitch was applied for flexible regulation of intracellular glycogen storage by controlling the expression of ADP-glucose pyrophosphorylase (glgC) [148]. The theophylline riboswitch was found to be operational in several other cyanobacterial species, including *Leptolyngbya* sp. BL 0902, *Nostoc* sp. 7120, and *Synechocystis* sp. WHSyn [149]. The theophylline riboswitch has also been used for chimeric riboswitch generation by combining it with a *Bacillus subtilis phuE* (adenine riboswitch). The chimeric riboswitch was validated in *Nostoc* sp. 7120 [150]. Overall, various synthetic biology toolboxes applicable to cyanobacteria are being developed to expand the pool of choice, which would contribute to effective engineering by enabling precise gene regulation of cyanobacteria.

### 4.2. Next-Generation Sequencing/Omics/Genome-Scale Model

Approximately 1500 cyanobacterial genome sequences have been registered in the NCBI genome database, and 196 of them, including the genome sequences of *Synechocystis* sp. PCC 6803 and *S. elongatus* PCC 7942, are completely assembled. In particular, the complete genome sequence of *Synechocystis* sp. PCC 6803 was reported as early as *E. coli* genome sequence, thus settling as a model organism among cyanobacteria [151]. Since then, with the development of diverse next-generation sequencing (NGS) techniques, various omics data such as transcriptome and translatome were generated based on the genome sequence [9]. Previously, transcriptome changes in response to stress conditions, such as temperature, light, and nutrition depletion, and effects of gene deletions were analyzed using cyanobacterium models, including *Synechocystis* sp. PCC 6803, *S. elongatus* PCC 7942, and *Nostoc* sp. 7120 [9]. In addition to the model cyanobacteria, recent transcriptome studies are being conducted in various non-model cyanobacterium species as well (Table 3). For example, in *Euhalothece* living in a hypersaline habitat, various salt resistance-related genes, such as Na^+^ transporting multiple resistance and pH adaptation systems, and glycine betaine biosynthesis enzymes were highly upregulated [152]. In addition, differential RNA-seq revealed genome-wide transcription start sites (TSSs) in *S. elongatus* UTEX 2973 and two types of *Fischerella* strains, which were used to elucidate the differences in transcriptional regulation resulting in different phenotypes [153,154]. Additionally, the application of Ribo-seq to observe the translatome responses of *Synechocystis* sp. PCC 6803 under carbon starvation condition was reported in 2018 [155]. In order to observe the post-transcriptional responses in cyanobacteria, omics studies such as proteomics through liquid chromatography with tandem mass spectrometry (LC-MS/MS) analysis and metabolomics through gas chromatography-mass spectrometry (GC-MS) and ^13^C isotopically nonstationary metabolic flux analysis have been implemented [5,156]. The massive omics data generated from the sequencing techniques described above served as the basis for understanding the cyanobacterial system under various conditions and also aided in the development of cyanobacterial GEMs.

GEM is an in silico tool that is useful for explaining the entire metabolism of an organism based on its genomic information. Given the ability to predict cellular metabolic behavior under given conditions or constraints, GEMs are mainly used for designing metabolic engineering strategies. GEM is an iteratively evolving system mended by new input information starting from the prior draft. Thus, more precise GEMs can be constructed by gathering greater types and amounts of genome information. GEM has been witnessing significant advancements with continuous accumulation of massive omics data supplied by various NGS techniques. Among cyanobacteria, *Synechocystis* sp. PCC 6803 has been studied most extensively, starting with the central carbon metabolic reconstruction under heterotrophic, mixotrophic, or autotrophic conditions [157,158]. The *Synechocystis* sp. PCC 6803 GEM was recently updated with more detailed photosynthesis and electron transport chain data [159,160]. GEM of other cyanobacterial species, such as *Arthrospira platensis*, *Cyanothece* sp., *Nostoc* sp., *S. elongatus* UTEX 2973, *S. elongatus* PCC 7942, and *Synechococcus* sp., are being established and further enhanced [139]. Through in silico GEM simulation, the bottleneck step can be selected for optimal engineering to enhance the production of value-added biochemicals. In the near future, the continuously evolving GEM would grow more useful in the metabolic engineering of cyanobacteria.

## 5. Conclusions and Future Perspectives

Cyanobacteria have significant industrial value owing to their ability to generate energy from photosynthesis and to produce various secondary metabolites. However, several improvements are required for cyanobacteria to meet the industry-level expectations and to establish themselves as a potential bioproduction platform. First, when using cyanobacterial native promoter or RBS, unexpected interaction may occur within the cell, which may reduce engineering efficiency. Therefore, development and application of the variety of orthogonal tools for engineering cyanobacteria is crucial. In addition, it is essential to obtain the precise metabolic network information to design strategies for the concise use of the synthetic biology tools. For example, the optimal production conditions can be discovered through promoter and RBS combination randomization, and the kind of neutral sites that can be used for chromosome integration can be expanded based on essential gene information found with transposon mutagenesis [143,146,211,212]. In addition, by applying the rapidly developing CRISPR application, it is possible to repress or activate multiple target genes at once, which can shorten the laborious and tedious engineering process caused by the polyploidy genome characteristic of cyanobacteria [101]. Systems biology enabled the discovery of various genetic tools by generating and accumulating massive omics data in cyanobacteria. In addition, development of GEM based on the accumulating omics and experimental data would lead to the development of a more accurate metabolic model.

## Figures and Tables

**Table 1:** Bioactive secondary metabolites produced in cyanobacteria.

Class	Metabolite	Bioactivity	Producing Species	Ref.
Terpene	Phycocyanin	Antioxidant, anti-inflammatory, neuroprotective, hepatoprotective	All cyanobacteria	[12,13,14,15,16]
Terpene	Carotenoids	Antioxidant, sunscreen	All cyanobacteria	[17,18]
Terpene	Squalene	Antioxidant	*Phormidium*	[19]
Alkaloid	Saxitoxin	Neurotoxin	*Anabaena*, *Aphanizomenon*, *Cylindrospermopsis*, *Lyngbya*, *Planktothrix*,	[20,21,22]
Indole	Nostodione	Antifungal	*Nostoc*	[23]
Indole alkaloid	Scytonemin	Anti-inflammatory, sunscreen	*Scytonema*, *Nostoc*	[24,25,26,27]
Indole alkaloid	Hapalindole	Antibacterial, anti-tuberculosis, anticancer	*Hapalosiphon*	[28,29]
Alkaloid/Polyketide synthase (PKS)	Anatoxin-a	Neurotoxin, anti-inflammatory	*Anabaena*, *Aphanizomenon*, *Cylindrospermum*, *Oscillatoria*, *Planktothrix*	[30,31]
Alkaloid/PKS	Aplysiatoxin	Cytotoxin, antiviral	*Moorea*	[32,33]
Alkaloid/Non-ribosomal peptide synthetase (NRPS)	Lyngbyatoxin	Cytotoxin, dermatotoxin	*Moorea*	[34]
Alkaloid/PKS-NRPS	Cylindrospermopsin	Cytotoxin	*Aphanizomenon*, *Cylindrospermopsis*, *Oscillatoria*, *Raphidiopsis*	[35,36,37]
PKS	Fischerellin	Antifungal, antialgal, anti-cyanobacterial	*Fischerella*	[38]
NRPS	β-N-methylamino-l-alanine	Neurotoxin	*Anabaena*, *Nostoc*	[39]
NRPS	Cyanopeptolin	Protease inhibitor	*Planktothrix*, *Microcystis*	[40,41]
PKS-NRPS	Microcystin	Hepatotoxin	*Microcystis*, *Nostoc*, *Planktothrix*, *Anabaena*	[40,42,43,44,45]
PKS-NRPS	Nodularin	Hepatotoxin	*Nodularia*	[46]
PKS-NRPS	Apratoxin	Anticancer	*Lyngbya*	[47]
PKS-NRPS	Aeruginoside	Protease inhibitor	*Planktothrix*	[48]
PKS-NRPS	Aeruginosin	Protease inhibitor	*Microcystis*, *Planktothrix*	[40,49]
PKS-NRPS	Cryptophycins	Cytotoxin	*Nostoc*	[50]
PKS-NRPS	Nostophycins	Cytotoxin	*Nostoc*	[51]
PKS-NRPS	Curacins	Cytotoxin	*Moorea*	[52]
PKS-NRPS	Hectochlorin	Cytotoxin	*Moorea*	[53]
PKS-NRPS	Jamaicamides	Neurotoxin	*Moorea*	[54]
PKS-NRPS	Dolastatin	Cytotoxin, anticancer, antiprotozoal	*Moorea*, *Lyngbya*, *Symploca*	[55,56]
Lipopeptide	Antillatoxin	Neurotoxin	*Moorea*	[57]
Lipopeptide	Carmabin	Antimalarial, anticancer, antiproliferative	*Moorea*	[58,59]
Lipopeptide	Lyngbyabellin	Cytotoxin, antifungal	*Moorea*, *Lyngbya*	[60,61]
Lipopeptide	Kalkitoxin	Neurotoxin	*Moorea*	[57]
Ribosomally synthesized and post-translationally modified peptide (RiPP)	Patellamide	Moderate cytotoxicity	*Prochloron*	[62]
RiPP	Microviridin	Protease inhibitor	*Microcystis*, *Planktothrix*	[63,64]
RiPP	Shinorin	Sunscreen	*Anabaena*, *Nostoc*	[65]
Fatty acid amide	Besarhanamide A	Moderate toxicity to brine shrimp	*Moorea*	[66]
Fatty acid amide	Semiplenamide	Toxicity to brine shrimp	*Lyngbya*	[67]
Lipopolysaccharide	Lipopolysaccharides	Endotoxin	All cyanobacteria	[68]
Polysaccharide	Polysaccharide	Antitumor, antiviral, antibacterial, anti-inflammatory, immunostimulant	All cyanobacteria	[69,70,71]
Nucleoside	Toyocamycin	Antifungal	*Tolypothrix*	[72]
Nucleoside	Tubercidin	Antifungal	*Tolypothrix*	[73]

**Table 2:** Recent studies of engineering cyanobacteria for biochemical production.

Strategy ^1^	Strain	Target ^2^	Gene	Ref.
HR	*S. elongatus* PCC 7942	Isoprene	*ispGS*, *idi*, *dxr*	[6]
HR	*S. elongatus* PCC 7942	Succinate *	*ppc*, *gltA*, *kgd*, *gabD*	[108]
HR	*S. elongatus* PCC 7942	Amorpha-4,11-diene, Squalene *	*dxs*, *idi*, *ispA*, *dxr*	[103]
HR	*S. elongatus* UTEX 2973	Sucrose *	*cscB*	[109]
HR	*Synechocystis* sp. PCC 6803	Isoprene	*ispS*	[110]
HR	*S. elongatus* PCC 7942	Isopropanol *	*sadh*, *thl*, *atoAD’*, *adc*	[111]
HR	*Synechocystis* sp. PCC 6803	Geranyllinalool	*NaGLS*	[112]
HR	*S. elongatus* PCC 7942	Squalene *	*dxs*, *idi*, *ispA*, *SQS*	[104]
HR	*S. elongatus* PCC 7942	Butyrate	*phaBJ*, *Ptb*, *buk*, *pte2*, *tesB*, *yciA*	[113]
HR	*Anabaena* sp. PCC 7120	Ethanol	*pdc*, *adhA*, *sigE*, *ald*, *invAB*	[114]
HR	*S. elongatus* PCC 7942	Sucrose *	*cscB*, *sps*, *glgC*	[115]
HR	*S. elongatus* PCC 7942	3-Hydroxybutyrate	*phaAB*, *tesB*, *nphT7*, *pptesB*, *yciA*, *pte1*	[116]
HR	*S. elongatus* PCC 7942	Heparosan	*galU*, *PmHS2*	[117]
HR	*Synechocystis* sp. PCC 6803	1-Butanol	*phaAB*, *nphT7*, *fadB*, *phaJ*, *ccr*, *ter*, *pduP*, *mhpF*, *yqhD*, *yjgB*, *pk*, *pta*, *adh*, *sigE*	[97]
HR	*Synechocystis* sp. PCC 6803	Sorbitol	*s6pdh*, *fbp*, *pnt*, *had1*, *had2*	[118]
HR	*Synechocystis* sp. PCC 6803	β-Phellandrene *	*GPPS*, *PHLS*	[119]
HR	*S. elongatus* PCC 7942	Acetone	*pdc*, *ald6*, *acs*, *pps*, *ppc*, *mmc*	[120]
HR	*S. elongatus* PCC 7942	Xylitol	*xylEFGH*, *XDH*, *DI*, *XR*	[121]
HR	*S. elongatus* PCC 7942	Trehalose *	*tpsp*, *Tret1*, *mts*, *glgCX*, *cscB*, *mth*	[122]
HR	*S. elongatus* PCC 7942	2,3-Butanediol	*alsD*, *alsS*, *adh*, *galP*, *zwf*, *edd*, *pgi*, *gnd*, *pfk*, *eda*, *cp12*, *rbcLXS*, *prk*	[5]
HR	*S. elongatus* PCC 7942	α-Farnesene	*AFS*	[123]
HR	*Synechocystis* sp. PCC 6803	Ethanol	*eno*, *pgk*, *pyk*, *prk*	[124]
HR	*S. elongatus* PCC 7942	Limonene *	*ls*, *GPPS*, *dxs*	[125]
HR	*Synechococcus* sp. PCC 7002	d-Lactate	*acsA*	[126]
epi	*Synechocystis* sp. PCC 6803	Isoprene	*ispS*	[127]
epi	*Synechocystis* sp. PCC 6803	p-Hydroxyphenylacetaldoxime, dhurrin	*CYP71E1*, *CYP79A1*, *UGT85B1*	[128]
epi	*Anabaena* sp. PCC 7120	Lyngbyatoxin A *	*ltxA-C*, *ltxA-D*	[129]
epi	*Synechocystis* sp. PCC 6803	Ethanol	*pdc*, *adh*, *rbcSC*, *70glpX*, *tktA*, *fbaA*	[98]
epi	*Synechocystis* sp. PCC 6803	Shinorine *	*FsABCD*, *APPT*	[130]
epi	*S. elongatus* UTEX 2973	Hapalindole *	*famH1*, *famH2*, *famH3*, *aph3*, *famE2*, *famD2*, *famC1*, *famC2*, *famC3*	[131]
HR + epi	*Synechocystis* sp. PCC 6803	Astaxanthin*	*crtWZ*, *dxs*, *idi*, *ispA*, *F/SBPase*, *RuBisCO*, *rpe*, *tktA*, *psy*	[107]
HR + epi	*S. elongatus* PCC 7002	2,3-Butanediol	*alsDS*, *adh*	[132]
HR + epi	*Synechocystis* sp. PCC 6803	Isobutanol	*kivd*, *adh*	[133]
HR + epi	*Synechocystis* sp. PCC 6803	Limonene *	*lims*, *rpi*, *rpe*, *GPPS*	[134]
CRISPR	*S. elongatus* PCC 7942	Succinate *	*glgC*, *gltA*, *ppc*	[135]
CRISPR + epi	*Synechocystis* sp. PCC 6803	Fatty alcohol *	*Maqu2220*, *DPW*, *plsX*, *aar*, *ado*, *sll1848*, *sll1752*, *slr2060*	[136]
CRISPR	*Synechocystis* sp. PCC 6803	*N*-Butanol, ethanol	*adhA*, *pdc*, *pduP*, *phaJ*, *ter*, *phaBCE*, *nphT7*, *sth*, *yqhD*, *xfpk*, *PL22*, *SAS2203*, *gltA*, *odhB*, *ackA*, *pyrF*, *nrtA*, *ndhD*	[99]
CRISPR	*S. elongatus* PCC 7942	Squalene *	*acnB*, *cpcB2*	[105]

^1^ HR, homologous recombination; epi, episomal expression ^2,^ * cyanobacterial natural product.

**Table 3:** Recent advances in omics studies of cyanobacteria.

Year	Omics Study	Strain	Ref.
2016	Genome-scale model (GEM) + Metabolome	*Synechococcus* sp. PCC 7002	[161]
2016	Metabolome	*S. elongatus* PCC 7942	[6]
2016	Metabolome + Transcriptome	*Synechocystis* sp. PCC 6803	[127]
2016	Proteome	*S. elongatus* PCC 7942	[162]
2016	Proteome	*Synechocystis* sp. PCC 6803	[163]
2016	Transcriptome	*S. elongatus* PCC 7942	[164]
2016	Transcriptome	*Synechocystis* sp. PCC 6803	[165]
2016	Transcriptome	*Prochlorococcus* NATL2A	[166]
2016	Transcriptome	*Nostoc* sp. PCC 7120	[167]
2016	Transcriptome	*S. elongatus* PCC 7942	[168]
2016	GEM	*S. elongatus* PCC 7942	[10]
2016	Transcriptome	*M. aeruginosa*	[169]
2017	Metabolome	*Synechococcus* sp. PCC 7002	[170]
2017	Metabolome	*S. elongatus* PCC 7942	[171]
2017	Metabolome	*S. elongatus* PCC 7942	[172]
2017	Metabolome	*S. elongatus* PCC 7942	[5]
2017	Metabolome	*Synechocystis* sp. PCC 6803	[173]
2017	Proteome	*Synechocystis* sp. PCC 6803	[174]
2017	Proteome	*Synechocystis* sp. PCC 6803	[175]
2017	Proteome	*Synechocystis* sp. PCC 6803	[176]
2017	Proteome	*Synechococcus* strains	[177]
2017	Proteome	*Prochlorococcus* strains	[178]
2017	Proteome	*P. marinus* SS 120	[179]
2017	Proteome	*Synechocystis* sp. PCC 6803	[180]
2017	Transcriptome	*Synechocystis* sp. PCC 6803	[181]
2017	Transcriptome + Interactome	*Synechocystis* sp. PCC 6803	[182]
2017	Transcriptome + Metabolome	*Synechococcus* sp. IU 625	[183]
2017	Transcription start site (TSS)	*F. muscicola* PCC 7414 and *F. thermalis* PCC 7521	[154]
2017	GEM	*Synechocystis* sp. PCC 6803	[184]
2017	GEM	*Nostoc* sp. PCC 7120	[185]
2017	GEM	*S. elongatus* UTEX 2973	[186]
2017	GEM	*Synechococcus* sp. PCC 7002	[161]
2018	Transcriptome	*M. aeruginosa*	[187]
2018	Transcriptome + Translatome	*Synechocystis* sp. PCC 6803	[155]
2018	TSS	*S. elongatus* UTEX 2973	[153]
2018	GEM	*Synechocystis* sp. PCC 6803	[161]
2019	Metabolome	*Synechococcus* sp. PCC 7002	[188]
2019	Proteome	*Synechocystis* sp. PCC 6803	[189]
2019	Transcriptome	*Prochlorococcus* MIT9313	[190]
2019	Transcriptome	*N. punctiforme* PCC 73102	[191]
2019	Transcriptome	*Leptolyngbya* sp. PCC 6406	[192]
2020	GEM	*Synechocystis* sp. PCC 6803	[160]
2020	Metabolome	*S. elongatus* PCC 11802 and PCC 11801	[193]
2020	Metabolome	*Nostoc* sp. UIC 10630	[194]
2020	Metabolome	*Leibleinia gracilis*	[195]
2020	Metabolome	*Synechocystis* sp. PCC 6803	[196]
2020	Metabolome	*S. elongatus* UTEX 2973	[197]
2020	Metabolome	*S. elongatus* PCC 11801	[198]
2020	Metabolome	*M. aeruginosa* PCC 7820 and PCC 7806	[199]
2020	Metabolome	*Synechocystis* sp. PCC 6803	[200]
2020	Metabolome	*Nodularia spumigena*	[201]
2020	Proteome	*Nostoc* sp. PCC 7120	[202]
2020	Proteome	*Synechococcus strains*	[203]
2020	Proteome	*Nodosilinea* strains	[204]
2020	Transcriptome	*Nostoc* sp. PCC 7120	[205]
2020	Transcriptome	*Euhalothece* sp. Z-M001	[152]
2020	Transcriptome	*Synechocystis* sp. PCC 6803	[206]
2020	Transcriptome	*N. punctiforme* PCC 73102	[207]
2020	Transcriptome	*Synechococcus* sp. PCC 7002	[208]
2020	Transcriptome + Metabolome	*Synechocystis* sp. PCC 6803	[107]
2020	GEM	*Synechococcus* sp. BDU 130192	[209]
2020	GEM	*A. variabilis* ATCC 29413	[210]
